# Bladder and Bowel Dysfunction Rehabilitation in Children with Acquired Brain Injury

**DOI:** 10.3390/children11111382

**Published:** 2024-11-14

**Authors:** Rita Chiminello, Chiara Pellegrino, Noemi Deanesi, Giulia Barone, Ida Barretta, Gaia Paolella, Maria Luisa Capitanucci, Antonio Maria Zaccara, Maria Laura Sollini, Giacomo Esposito, Donatella Lettori, Gessica Della Bella, Enrico Castelli, Giovanni Mosiello

**Affiliations:** 1 Neurorehabilitation Unit, Bambino Gesù Children’s Hospital, IRCCS, 00165 Rome, Italy; rita.chiminello@opbg.net (R.C.); enrico.castelli@opbg.net (E.C.); 2 Division of Neuro-Urology, Bambino Gesù Children’s Hospital, IRCCS, 00165 Rome, Italyantoniomaria.zaccara@opbg.net (A.M.Z.); giovanni.mosiello@opbg.net (G.M.); 3 Department of Urology, Fondazione Policlinico Universitario Campus Bio-Medico di Roma, 00128 Rome, Italy; 4 Department of Neuroscience, Rehabilitation, Ophthalmology, Genetics and Maternal and Child Sciences (DINOGMI), University of Genoa, 16132 Genoa, Italy; 5 Clinical Science and Translational Medicine, Tissue Engineering and Remodeling Biotechnologies for Body Function PhD School, University of Rome Tor Vergata, 00133 Rome, Italy; 6 Neurosurgery Unit, Bambino Gesù Children’s Hospital, IRCCS, 00165 Rome, Italy; 7 Neurorehabilitation and Adapted Physical Activity Day Hospital, Bambino Gesù Children’s Hospital, IRCCS, 00165 Rome, Italy; gessica.dellabella@opbg.net

**Keywords:** neurogenic bladder dysfunction, neurogenic bowel dysfunction, cerebral palsy, acquired brain injury, pediatric urology, continence

## Abstract

**Objective:** To evaluate neurogenic bladder and bowel dysfunction (NBBD) in children with cerebral palsy (CP) and acquired brain injury (ABI), a condition considered less frequent in those patients than in children with spinal cord injury (SCI), and to study the relationship between NBBD and disability grade in this population. **Study Design:** We retrospectively reviewed the clinical data of all patients (aged 3–18 years old) admitted during a three-month observation in our neurorehabilitation department. Data collected were as follows: demographic parameters; disability status (Wee-FIM Scale, Gross Motor Function Classification System (GMFCS) and the Communication Function Classification System); and gastrointestinal and urological symptoms (diaries, Bristol scale, Pad Test and International Consultation on Incontinence Modular Questionnaire). **Results:** Sixty patients were enrolled (31 females, 29 males): 30 CP, 17 ABI, 3 SCI, and 10 others with neurological diseases. All presented urinary incontinence without gender differences. CP and ABI had major incidences of bowel dysfunction (50% and 64.7%, respectively) and SCI of urinary tract infections (66.6%) and enuresis (100%). A major incidence of symptoms was recorded in patients with higher GMFCS levels (level 3-4-5). **Conclusions:** NBBD has a high frequency in children with CP and ABI, as in SCI. More attention is needed from pediatricians and pediatric urologists for this clinical entity. Further studies are needed to better understand clinical relevance and, therefore, to establish specific management.

## 1. Introduction

Neurogenic bladder and bowel dysfunction (NBBD) in children with spina bifida is a well-known pathological condition, as well as in other conditions such as spinal dysraphism in anorectal malformation, oncological diseases, and spinal cord injury (SCI) [1,2,3,4,5,6,7]. Cerebral palsy (CP) and acquired brain injury (ABI) are commonly observed in a pediatric neurorehabilitation setting, where the onset of more serious physical conditions and the relevance of bladder and bowel dysfunction are often diminished [8,9].

CP is the most frequent neurological condition, with an incidence of 1.5–3.5 per 1000 births. In CP, the location and degree of neurological damage define the severity of the disease (e.g., motor and mental impairment, communication barrier, hearing or vision difficulties, seizures). Children with CP experience a considerable prevalence of NBBD, manifesting with urinary incontinence, enuresis, urinary urgency or frequency, encopresis, constipation, and lower abdominal and pelvic pain [1,10,11,12].

ABI is described as a neurological insult that occurs after normal brain development, and it is increasingly responsible for neurological disabilities. ABI can be caused by heterogeneous etiologies, including trauma, which is the most frequent form, or non-traumatic cause (e.g., vascular malformation, infection). ABI can lead to complex clinical patterns engaging physical, intellectual and social spheres where a different grade of recovery may occur [13,14].

ABI and CP represent a clinical challenge where different pathological patterns can be present, including different grades of gross motor impairment and respiratory failure and impairment of sensory and/or motor pathways to the lower urinary tract, rectum, and anal sphincter, leading to dysfunction in urinary and gastrointestinal systems [7].

The aim of our study was to ascertain the prevalence of NBBD among patients with ABI and CP and to explore their correlation with the severity of the respective injuries.

## 2. Materials and Methods

All new patients admitted to our neurorehabilitation department located in a third-level Children’s Hospital and Research Institute, which is identified as a high specialization center by the European Reference Network ITHACA and EUROGEN, during three months of observation and aged 3 to 18 years old were evaluated. Data collected were clinical diagnosis and demographic parameters; disability grade evaluated through specific tests (Wee-FIM^®^, GMFCS and CFCS); and gastrointestinal and urological status assessed through the compilation of daily diaries, Bristol scale, Pad Test, ICIQ and bladder emptying monitoring.

The tests used are explained in more detail below:

- The Wee-FIM^®^ functional ability 18-item questionnaire was used to evaluate change in functional status for children aged 6 months–7 years and patients > 7 years with disabilities and delays in functional development. It clarifies self-care (8 items) and functional (5 items) and cognitive (5 items) abilities [15].
- Gross Motor Function Classification System (GMFCS) is a clinical tool to categorize 5 different levels of gross motor skills for children aged 6–12 years. The system extends from Level I, in which the patient can perform gross motor functions with limitations in speed, balance and coordination, up to Level V, with limited antigravity control of the head and trunk and the need for wheelchair transport [16].
- Communication Function Classification System (CFCS) was used to evaluate individual communication, dividing the effectiveness of communication into five levels, from Level I, with no communication problems with family members and non-family members, up to Level V, with rare effective communicative interactions, even with familiar people [17].
- Bristol scale is used to define bowel habits as normal or altered, ranging from Type 1 (with increased consistency of stools, “like nuts”) to Type 7 (completely liquid stools); a visual scale of the different types of stools was presented to patients or their caregivers [18].
- Pad Test is a non-invasive diagnostic test that can quantify urinary leakage by weighing a pad before and after its use during a 24 h period of normal activity [19].
- International Consultation on Incontinence Modular Questionnaire (ICIQ) is a questionnaire to assess urinary incontinence or other urinary symptoms [20].
- Bladder emptying monitoring was carried out by evaluating the bladder filling volume before and after micturition/urine leakage using an Ultrasound Bladder Scan, which allows for easy evaluation, even at the patient’s bedside [21].

All patients underwent a multidisciplinary diagnostic and therapeutic approach, including evaluations by the neurologist, physiatrist, neurorehabilitation specialist, physiotherapist, pediatric urologist and pediatric digestive surgeon.

All data were collected after a written consent form was signed by parents. All instruments (questionnaires, diaries, etc.) are commonly used in our daily clinical practice, and for this reason, no specific authorization was required for their use. Anyway, the retrospective evaluation and publication of data was authorized by our Institution.

Data were analyzed using MedCalc^®^ Statistical Software version 23.0.2 (MedCalc Software Ltd., Ostend, Belgium; https://www.medcalc.org; accessed on 12 May 2024).

Analysis for normally distributed continuous variables was performed using analysis of variance (ANOVA). The *p* values reported for the ANOVA are the values for the specific factor of interest. Categorical variables were analyzed using the Fisher’s Exact test; *p* < 0.05 (two-sided) was considered statistically significant.

## 3. Results

During the study period, 64 patients were evaluated to be enrolled, and 4 of them were excluded: 3 families refused to participate in the study, and 1 did not complete all the tests requested.

In total, 60 patients were enrolled: 31 females and 29 males with an average age of 8 years (interquartile: 3 years–17.5 years).

Of these 60 patients, 30 presented with CP, 17 with ABI, 3 with SCI and 10 others with neurological diseases, as follows: 1 Adams–Oliver Syndrome with encephalocele, 1 Ataxia, 1 Rubinstein–Taybi Syndrome, 1 congenital disorders of glycosylation, 1 Williams Syndrome and 5 undefined neurodevelopmental disorders on genetic study.

The results of all tests applied are summarized in Table 1.

At the first urological evaluation, before admission, all patients presented with at least one type of incontinence, and no differences between sexes were observed (*p* = 1).

Patients with SCI had no significant differences compared with other groups of patients in terms of frequencies of urinary tract infections (UTIs), enuresis (defined as involuntary urination during the night), daytime urinary incontinence and dysfunctional voiding with urinary retention managed with clean intermittent catheterization.

Patients with CP and ABI had a major incidence of bowel dysfunction: encopresis was present in 50% of CP patients (15 patients) and 64.7% of ABI patients (11 patients); constipation was present in 43.3% (13 patients) of CP patients and 52.9% (9 patients) of ABI patients; *p* = 0.03.

All patients underwent an assessment of disability grade.

**Figure 1:**
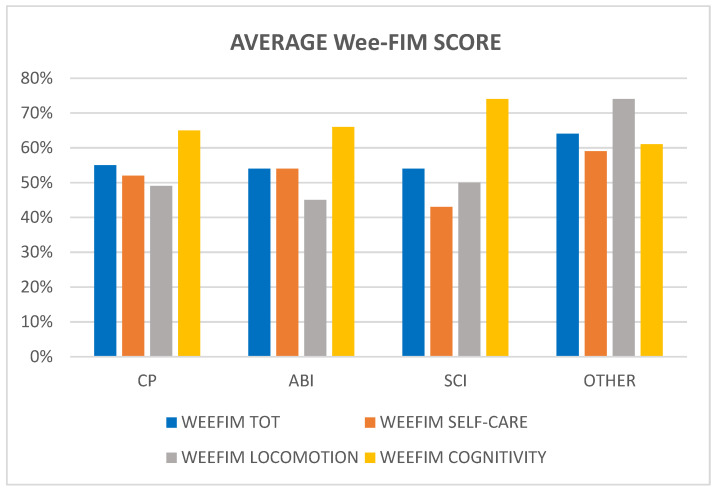
Average Wee-FIM scores divided by pathology (*p* value for operation in ANOVA).

The average Wee-FIM scores (Figure 1) are slightly similar for each group (*p* = 0.9). Patients with SCI seemed to show a minor self-care autonomy (43%) but a higher score in the cognitive area (74%), although the results were not statistically significant (*p* = 0.74); patients with other neurological pathologies obtained the highest score in the locomotion area (74%, *p* value 0.004).

**Figure 2:**
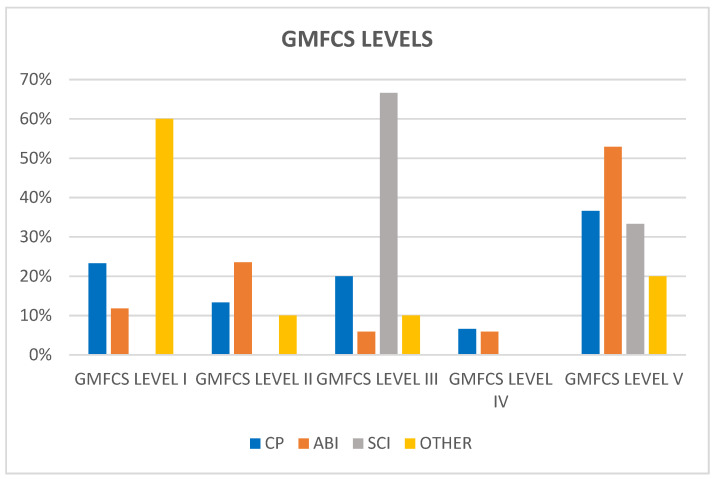
Distribution of GMFCS levels for each group of patients.

Regarding GMFCS level distribution (Figure 2), most patients with CP and ABI fall under GMFCS level V (36.6% CP and 52.9% ABI), whilst most patients with SCI fall under GMFCS level III (66.6%). According to the results of Wee-FIM scores, patients with other neurological pathologies showed better gross motor function (60% were included in GMFCS level I).

**Figure 3:**
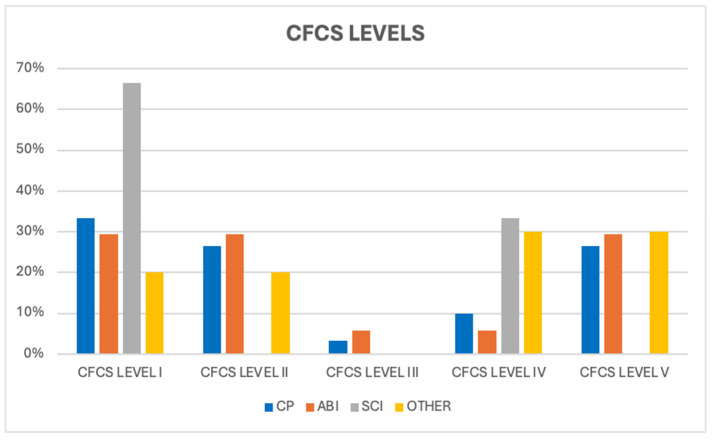
Distribution of CFCS levels for each group of patients.

Patients with ABI and CP were distributed almost equally under CFCS level I (29.4% and 33.3%, respectively) and level V (29.4% and 26.6%, respectively) (Figure 3).

Using the GMFCS scale for the generic representation of disability grade, we evaluated the incidence of urinary and gastrointestinal symptoms at each level to see if a correlation between them could be defined. As explained in more detail in Table 2, we compared the incidence of urological and bowel symptoms with different GMFCS levels: we found a statistically significant correlation between enuresis and urgency with higher levels of GMFCS score; *p* = 0.006 and *p* = 0.001, respectively.

## 4. Discussion

Neurogenic bladder and bowel dysfunction are a clinical challenge in children with neurological pathologies, with a wide spectrum of manifestations relating to anatomical level and the type and degree of the malformation/lesion. The presence of NBBD can further worsen the quality of life of these patients and their parents; it requires multidisciplinary management and targeted therapy to obtain the best results for the individual patient.

### 4.1. Therapeutic Approach

The presence of neurological lesions affecting the brain, spinal cord and somatic peripheral nervous system can lead to a well-known clinical condition called neurogenic bladder and neurogenic bowel. Major attention is commonly reserved for bladder dysfunction with respect to bowel because a progressive worsening of bladder function can cause recurrent UTIs, urinary incontinence, and renal damage, up to chronic renal failure. Therefore, it is necessary to promptly identify bladder dysfunction and proceed with its therapeutic approach. This may be based on the use of behavioral rules, clean intermittent catheterization pharmacological therapy (such as anticholinergic drugs), intratradetrusor injection of Onabotulinum Toxin-A or the use of surgical techniques (such as the creation of vesicostomy, suprapubic catheter/button cystostomy placement, bladder augmentation). Therapy must be modulated based on the patient’s general clinical status and comorbidities, with intellectual disability mainly guiding the most appropriate therapeutic choice [22,23,24].

### 4.2. Continence Achievement

The existing literature suggests that continence achievement is often delayed or not achieved at all in these children. This aspect is explained by the fact that the bladder filling and emptying phases are regulated by the autonomic and somatic nervous system, including cortical pathways, with the micturition center localized in the brainstem; all these structures and pathways may be altered by their injuries/malformations. In the general population, the capacity for voluntary emptying of the bladder or its postponement is achieved between the second and third years of life, with complete urological control normally reached by the fourth year of life. ABI and CP can interfere in this process of bladder maturation and continence achievement. Wright et al. found that, among patients with bilateral CP at 17 years of age, daytime continence was achieved in 60.8%, while night-time continence in 54.6%; moreover, among continent patients, this was reached at the age of 5.5 years in 86% of all the patients [11,25,26]. This delay could be attributed to various factors, such as the incomplete development of bladder or sphincter control, reduced awareness and control of detrusor contraction, poor coordination of pelvic floor muscles during urination and defecation, altered perception of bowel movements, constipation, and cognitive and communicative disability.

### 4.3. Hints of Urodynamics

From a urodynamic point of view, neurogenic detrusor overactivity (NDO), reduced cystometric capacity and detrusor–sphincter dyssynergia (DSD) are common conditions among children with CP. NDO is found in approximately 59–70% of cases; this frequent finding can be explained by the suprapontine lesions present in patients with CP, for example, which lead to an elimination of inhibitory control over the pontine micturition center and the onset of NDO. Consequently, NDO causes a small bladder capacity compared to the age standard (a prognostic factor for incontinence) and involuntary bladder contractions (with possible urine leakage and/or renal damage). Additionally, children with spasticity often present with overactivity of the pelvic floor muscles, leading to coordination issues between bladder contraction and pelvic floor muscle relaxation. DSD can cause increased post-void residual, a predisposition factor for UTIs. All these patterns can lead to urinary incontinence, vesico-ureteral reflux and recurrent UTIs [1,9,27].

### 4.4. Association Between BBD and Motor Function Level

Furthermore, previous studies have identified correlations between the age at which continence is attained, cognitive and communication abilities and motor function level. Children with higher motor function levels tend to achieve continence at an earlier age, while cognitive ability and intellectual impairment also play a significant role, and a combination of cognitive and motor impairment is associated with a high prevalence of NBBD. Some authors found that patients with intellectual impairment and spastic quadriplegia were incontinent with higher frequency than subjects with high intellectual ability and hemi-/diplegia; they concluded that the combination of spasticity and intellectual impairment influenced continence more than how the single factors do independently [9,11,12,26,28,29,30,31,32]. Consistent with these findings, our assessment of injury severity using the GMFCS revealed a clear correlation between higher levels of injury severity (levels 3-4-5) and increased frequency of bladder and bowel dysfunction. This correlation highlights the impact of neurological injury severity on the onset and severity of NBBD.

### 4.5. Urological Sphere

Notably, all patients included in our study exhibited at least one type of incontinence, underlining the high frequency of urological dysfunction in these children. We observed daytime urinary incontinence rates of 26.6% in CP and 11.7% in ABI patients. Furthermore, it was also highlighted how decreased urinary stream may increase with the patient’s age, as reported by Yokoyama et al. [33] in their study of 132 patients with CP aged between 5 and 59 years (mean age of 23.2 years); however, the frequency of urinary incontinence was comparable across all age groups. UTIs are another frequent finding in these patients, affecting one-fifth of CP patients, with a complex multifactorial origin due to bladder dysfunction, post-void residue and vesico-ureteral reflux but also constipation, low fluid intake and possible decrease in hygiene. Some authors have suggested that asymptomatic bacteriuria in neurological patients under clean intermittent catheterization without evidence of vesicoureteral reflux is not associated with renal scarring and does not need antibiotic therapy [12,24,27,34,35].

### 4.6. Bowel Sphere

As for bladder control, regular bowel emptying can also be altered by lesions of the nervous system (sensory and/or motor alterations), leading to “neurogenic bowel”, where constipation and fecal incontinence often coexist. As already mentioned for the bladder, even bowel control is usually achieved between 2 and 4 years of age; disabled patients reach this step at a higher age. Correct management of bowel dysfunction is necessary, starting from behavioral advice and diet to the use of osmotic laxatives and enemas, up to the use of trans-anal irrigation, which can reduce fecal leakage with a beneficial effect on sphincter tone and rectal volume, or surgical procedure (such as Malone antegrade continence enema or colostomy creation) [1,11,23].

According to previous studies, bowel problems are widely diffuse in neurologic patients, with 39.2–54% of children with CP suffering from encopresis and 26–74% from constipation. The type, degree and level of the neurological lesion can lead to different functional changes in intestinal functioning, resulting in various clinical manifestations that often overlap with each other. In our study, the incidence of bowel dysfunction was high, with encopresis revealed in 50% of CP patients and 64.7% of ABI patients; constipation was present in 43.3% of CP patients and 52.9% of ABI patients.

This research sheds light on an underreported aspect of neurological disorders in children, emphasizing the need for increased awareness and research in this area. Understanding the prevalence and severity of NBBD in CP and ABI patients is crucial to developing targeted interventions and improving their quality of life. Furthermore, it highlights the necessity of comprehensive healthcare for these patients, addressing not only their primary condition but also the associated complications to provide a comprehensive approach [36].

## 5. Limits of the Study

Limitations of the study are as follows: single-center experience with a short timeframe of data collection; small number of patients collected and no uniformity of the categories analyzed; no quality-of-life questionnaire of patients and caregivers; no data reporting was used for NBBD patients’ management according to the severity of disabilities and patients’ mental impairment.

## 6. Conclusions

Neurogenic bladder and bowel dysfunction have a high frequency in children with ABI and CP. While NBBD is commonly considered in patients with spina bifida and other dysraphisms and SCI, according to our results, major attention by pediatricians and pediatric urologists must be paid to NBBD in ABI and CP, either due to the high frequency or the increasing proportion of the population affected by ABI and CP that is observed in pediatric hospitals and neurorehabilitation units. A multidisciplinary approach seems mandatory and, in our opinion, could be useful for standardizing the treatment of NBBD in CP and ABI, considering the fact that actual guidelines mainly refer to spina bifida patients, without considering the high degree of intellectual disability existing in these populations.

In this regard, it could be useful to increase the use of validated questionnaires, such as ICIQ, in all patients admitted to neurorehabilitation units.

This work is generated within the European Reference Network for Rare Urogenital Diseases and Complex Conditions (ERN EUROGEN).

## Figures and Tables

**Table 1:** Percentage of urinary and gastrointestinal symptoms in each group of pathology.

	CP (30 pts)	ABI (17 pts)	SCI (3 pts)	OTHER (10 pts)	*p*
UTI; *n*, (%)	4 (13.3)	4 (23.5)	2 (66.6)	0 (0)	0.1
ENURESIS; *n*, (%)	18 (60)	10 (58.8)	3 (100)	7 (70)	0.2
URGENCY; *n*, (%)	10 (33.3)	12 (41.2)	1 (33.3)	3 (30)	0.9
UI; *n*, (%)	8 (26.6)	2 (11.7)	1 (33.3)	3 (30)	0.6
DV; *n*, (%)	7 (23.3)	6 (35.2)	2 (66.6)	2 (20)	0.3
ENCOPRESIS; *n*, (%)	15 (50)	11 (64.7)	1 (33.3)	3 (30)	0.02
CONSTIPATION; *n*, (%)	13 (43.3)	9 (52.9)	0 (0)	5 (50)	0.03

*n*: number of patients; UTI: urinary tract infection; UI: urinary incontinence; DV: dysfunctional voiding; pts: patients.

**Table 2:** Correlation between disability’s grade according to GMFCS and urinary and gastrointestinal symptoms.

GMFCS	Level I (15 pts)	Level II 9 pts)	Level III (10 pts)	Level IV (3 pts)	Level V (23 pts)	*p*
UTI; *n*, (%)	2 (13.3)	1 (11.1)	2 (20)	0 (0)	5 (21.7)	0.4
ENURESIS; *n*, (%)	6 (40)	5 (55.5)	6 (60	2 (66.6)	19 (82.6)	0.006
URGENCY; *n*, (%)	7 (46.4)	4 (44.4)	6 (60)	2 (66.6)	19 (82.6)	0.001
UI; *n*, (%)	3 (20)	3 (33.3)	3 (30)	1 (33.3)	4 (17.4)	0.8
ENCOPRESIS; *n*, (%)	6 (40)	4 (44)	5 (50)	2 (66.6)	14 (60.8)	0.6
CONSTIPATION; *n*, (%)	5 (33.3)	4 (44.4)	3 (30)	2 (66.6)	10 (43.4)	0.4

UI: urinary incontinence; pts: patients.

## Data Availability

Patients’ data are reported in this paper. Other data are available in their clinical charts if necessary.

## Abbreviations

NBBD: neurogenic bladder and bowel dysfunction; SCI: spinal cord injury; CP: cerebral palsy; ABI: acquired brain injury; GMFCS: Gross Motor Function Classification System; CFCS: Communication Function Classification System; UTIs: urinary tract infections; NDO: neurogenic detrusor overactivity.
